# Novel Photonic Bio-Chip Sensor Based on Strained Graphene Sheets for Blood Cell Sorting

**DOI:** 10.3390/molecules26185585

**Published:** 2021-09-14

**Authors:** Fatemeh Ghasemi, Sepehr Razi

**Affiliations:** 1 Laser Center, Institute of Physical Chemistry, Polish Academy of Sciences, Kasprzaka 44/52, 01-224 Warsaw, Poland; 2 Optics and Laser Engineering Group, Department of Industrial Technologies, Urmia University of Technology (UUT), Urmia 57166-17165, Iran; s.razi@uut.ac.ir

**Keywords:** biosensor, photonic crystal, graphene, strain, label-free, tunable

## Abstract

A photonic biochip with a tunable response in the visible range is suggested for blood cell sorting applications. Multi-layers of ZnS and Ge slabs (as the main building blocks), hosting a cell in which bio-sample could be injected, are considered as the core of the sensor. In order to increase the sensitivity of the chip, the bio-cell is capsulated inside air slabs, and its walls are coated with graphene sheets. Paying special attention to white and red blood components, the optimum values for structural parameters are extracted first. Tunability of the sensor detectivity is then explored by finding the role of the probe light incident angle, as well as its polarization. The strain of the graphene layer and angle in which it is applied are also suggested to further improve the performance tunability. Results reflect that the biochip can effectively identify selected components through their induced different optical features, besides of the different figure of merit and sensitivity amounts that are recorded for them by the sensor.

## 1. Introduction

For more than two decades, remarkable efforts have been made to design and fabricate label-free, photonic chips for the fast detection of the key agents in biological solutions [1]. In between, due to their valuable features, such as fast real-time responses and remarkable sensitivities, transducers, based on surface waves have received more attention for such applications [2,3,4,5,6,7]. Sensors based on the plasmon resonances and Bloch surface waves (BSW) are the two most commonly considered ones at present [5,6,7,8,9,10,11]. In other words, as excitation/suppression of these waves is seriously sensitive to any small perturbation in the surrounding environment; they can easily reflect the characteristics of the existing ingredients in contact with the sensor, i.e., as each bio-agent has specific optical features, its coupling to a surface wave next to it would be different. Thus, the presence of a definite agent or change in its concentration might be detected, via its specific effect on these waves (such as specific change in intensity, phase, or polarization of the wave, as well as a shift in its central frequency or even the wave’s total suppression). Furthermore, it is proven that these surface waves are excited in different angles, for different bio-agents, in the case of Kreschman and Otto configuration-based optical biosensors [5,6,7]. Surface plasmon resonances (SPRs) are usually excited at the interface of metal-dielectric or metal-analyte layers [6,7,8,9]. However, now it has been well proven that the sensors based on SPR modes face numerous challenges, including difficulties in excitations of resonant modes and their short-term stabilities, having significant absorption losses, and thus, low sensitivity and complexities in the fabrication process, as well as optimum working for a limited extent of bio-agents [6,7,8,9,10,11]. As result, photonic crystals (PCs), under Kretschmann/Otto configurations that monitor alterations of the excited BSW modes, are undoubtedly more discussed at present [11,12,13]. The BSWs are electromagnetic waves that could be localized at the interface of a homogeneous and a periodic dielectric media, two adjacent photonic crystals (PCs), or a PhC and one left-handed metamaterial [10,11,12,13,14,15]. These waves decay exponentially along the normal direction, away from the surface into two surrounding layers. In these sensors, alternating slabs of (usually) nonmagnetic dielectric materials with different refractive indexes, form the core of the detector. An external light is coupled to the PC core through a prism of an appropriate refractive index that is usually placed on top of the sensor. The other side (exit side) of the sensor is in contact with the analyte that is going to be analyzed. Sensing takes place by considering the narrow resonances being created in the reflectance spectrum at a specific incident angle, which itself depends on the refractive index of the detected bio-agent, as well as the mode of the excited BSW resonance [12,13,14,15]. Detection of many bio-agents is possible in this way, as BSW resonances are spectrally tunable, having low absorption, and thus, can propagate to long lengths on the surface (interface between the sensor and bio-sample edge).

However, although BSW-based biosensors have been able to remove many limitations, they also suffer from some weaknesses that encourage scientists to find alternatives with less complexity in structures and even better responses in performance. In more detail, it is preferred to have a sensor with as few pieces as possible. The existence of any extra optical elements (such as the adjusting prism) not only increases the structure complexity and makes it difficult to use the sensor but also raises the fabrication cost. Furthermore, in most cases, it is necessary to apply bulk signal corrections for the received reflections in these sensors [13,14,15,16]. The necessity starts from the fact that, in these sensors, it is difficult to separate contributions of surface under study and the neighboring bulk layer in the detected signal. Of course, it is obvious that, due to the PC-based structure of BSW sensors, the amount of necessary corrections is much less than the amount needed in SPR sensors, but it still exists [13,14,15,16]. Another challenge in the performance of BSW sensors is the limited field penetration depths in the sample that, in turn, limit studying the internal parts of the sample. In this case, it is necessary to excite the s- and p-polarized modes simultaneously, which might have different penetration depths [16]. However, the implementation of this approach (simultaneous excitation of BSWs with different polarizations) is not that easy in practice. Other valuable approaches to overcome the limitations of the electromagnetic propagation depths have been suggested recently, in the appreciated references of [17,18].

In this manuscript, motivated by the existing challenges concerning the common bio-sensor configurations and the importance of the issue from application point of view, a novel photonic bio-chip, based on common one-dimensional PC (1D-PC), is suggested. It is made of a PC, including alternating layers of nonmagnetic slabs and a cell, which acts as a reservoir for a bio-fluid, i.e., its structure is not in Kretschmann or Otto’s configurations and does not include any metallic layer. Then, its performance will be far from any challenges that were discussed above. Furthermore, according to the numerous advantages that have been discovered for 2D-materials in last decades [19,20,21,22], walls of the considered bio-cell are covered by single sheets of graphene, to be benefited from the unique features of this amazing material. Details of the considered advantages are discussed in the next section. The structure is optimized in such a way as to be able to detect two key factors of red blood components (RBCs) and white blood components (WBCs), in very little volume of bio-fluid, at a visible frequency range. The presented bio-chip is not only different from the previously discussed sensors from structural configuration and ease of its fabrication and performance in practice, but also its optimum working frequency is in the range of visible light for which lots of sources are simply available. Additionally, as will be discussed extensively in the results section, its performance is highly tunable and is in a transmittance format that can give information of the whole bio-sample at two polarization states of transverse electric (TE) and transverse magnetic (TM).

It is also worth noting that the reason for selecting these specific blood components (i.e., WBC and RBC) to check the performance of the sensor is as follows: nowadays, blood analysis is widely implemented as one of the early detection methods for many diseases, such as heart problems, AIDS, hepatitis, and many types of cancers. In these diseases, certain proteins or antibodies are released in the blood streams and their detection might realize the detection of the related illness [3,23]. However, not only is the detection of novel ingredients in blood content demanded to identify any of these diseases but there also exists a serious necessity to well-monitor any changes in the main blood components themselves. In a general classification, blood includes three main cell types, named red blood components (RBC), plates, and white blood components (WBC) [23]. RBCs are responsible for the transportation of oxygen from the heart to the tissues and for transferring CO_2_ away. On the other hand, WBCs have the most important role in the body’s immune system. Then, any change in their concentration or classification might reflect the presence of infection or medical problems, as well as irregularities in metabolism [23]. Thus, it is important to focus on the design and fabrication of sensors that can monitor these important components.

This manuscript is organized as follows: details of the geometrical model of the bio-chip (besides the mathematical formulations used in theoretical modeling) are presented in the next section. Optical features of the chip are explored in Section 3, by studying the role of the structural parameters. Then, finding the optimum values of these parameters, potential of the structure for sensing applications is studied systematically in the same section, by taking into consideration of the effect of the probe light incident angle and its polarization. The issue is followed by studying how the detectivity of the bio-chip is dependent on the strain amount applied to the graphene layers and the orientation in which the strain is applied. Finally, the study is concluded by referring to the advantages of the structure, as well as its optimum response interval, to reflect how the suggested chip might find appropriate applications in biotechnology.

## 2. Structural Configuration and Theoretical Approaches

### 2.1. Geometrical Structure and Involved Parts

**Figure 1:**
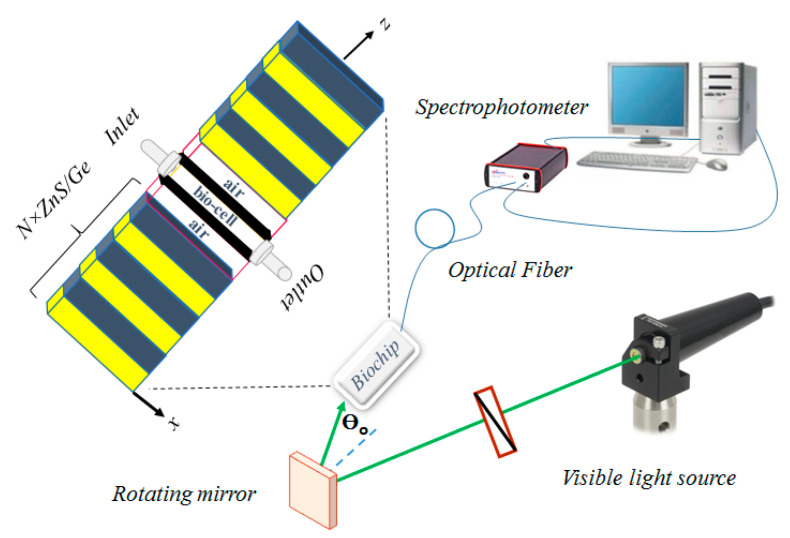
Schematic sketch of the suggested bio-chip and the involved parts in sensing procedures.

Details of the central core of the suggested photonic biochip and the other involved parts are schematically illustrated in Figure 1. As with any standard biosensor, there exists three main parts in this sensing system: the light source, core of the sensor, and analyzing section. The visible probe light passes through the appropriate polarization optics, to be polarized in a specific manner. In more detail, the probe light is transferred from a thin-film polarizer to be linearly polarized. Then, it is conducted to propagate through a phase retarder, in order to control the phase difference between the TE and TM components and reach any state of elliptical polarization. Light is then passed through the bio-chip, using a rotating mirror that can also tune the light incident angle on the sensor core. The light transmitted through the chip is captured by an optical fiber placed at the other side of the chip and conducted via the spectrophotometer, with which the transmittance spectrum might be recorded. As is shown in the 3D stereogram, in the same figure, the bio-chip is made of stacks of ZnS/Ge alternating layers, placed at two sides of the bio-cell, which were filled/emptied through nanofluidic channels. It is worth noting that deposition of Ge layers on ZnS slabs has been already performed successfully in experiments [24,25]. It has been shown that, when controlling the deposition time, it is possible to control the layer’s thickness [26]. Then, the suggested configuration could be easily implemented in practice. Thus, the heterostructure is considered as (ZnS/Ge)^N^ bio-cell (ZnS/Ge)^N^, where *N* is the repetition number of unit-cells stacked along the z-axis. Interfaces of the layers are considered parallel to the x-y-plane, and the z-axis is assumed to be normal for the structure. Surrounding the atmosphere is air, and the light incident angle is measured with respect to the z-axis. The thickness of the deposited layers might be determined simply using scanning electron microscope (SEM) imaging from a cross section of the structure. Another alternative, that can also provide information regarding the refractive index and extinction coefficient distribution inside the structure (and thus, the information of the layers thickness) is using the ellipsometric approach. However, this method has some more difficulties, as extra data analysis and fitting procedures are necessary after experimental measurements [27]. Two side walls of the bio-cell, that are along the probe light propagation direction, are covered by a graphene sheet, to benefit from the advantages of the 2D-materials. The number of sheets on each wall is just one, and their oxidation degree is considered to be zero. In experiment, the layers of graphene sheets could be deposited by using the valuable method presented in [28]. In this approach, Suk et al. have suggested a wet transfer method, to relocate chemical vapor deposition (CVD) grown monolayer graphene sheet onto a flat glass substrate at surface area as large as 1 cm × 1 cm. They have reported a transmittance amount of over 97% for the glass substrate covered by the monolayer of the graphene sheet @ λ = 550 nm (which is in the wavelength range being considered in our study).

### 2.2. Graphene Layer’s Role in Performance of the Suggested Sensor 

It is very well proven that one atom thick graphene sheets show many unique features, such as high optical transparency, low reluctance, and stable structure [17,18,19,20]. However, there are several other characteristics of graphene, as well as graphene oxide [29] that motivate researchers to benefit them in bio-sensor applications. Graphene has a nice biomolecule adsorbent efficiency, as is a two-dimensional (2D) material made of SP_2_-bonded carbon atoms settled in honeycomb lattice, which results in a high surface to volume ratio. Then, the strong adsorption of the biomolecules on its surface are possible via a π stacking interaction between its hexagonal cells and the carbon-based ring patterns, which are abundant in biomolecules [8,9,21]. Furthermore, graphene has a high response time when is illuminated by a light source, which is also very vital for bio-sensing objectives. Additionally, graphene is an extremely low-noise material that might be utilized as a capable layer in detecting low-weight particles (such as bio-agents) attached to its surface [30,31]. Combining these important features with the graphene’s ultrahigh, sensitive optical conductivity, even at room temperature (due to the massless Dirac charge carries), might reasonably realize the fabrication of a very potent sensor.

Paying attention to all these important points, it is clear that the considered monolayers of the graphene sheets in the suggested structure can make an impressive contribution to its well performance. The presence of graphene layers in our structure provides an appropriate substrate for the considered blood components (i.e., RBC and WBC) to be adsorbed. Thus, their coupling with the probe light might be realized more effectively and the biosensor sensitivity will be increased. In more detail, when such molecules are attached to the surface of the graphene sheets, the occupation of the charge carrier density states will be changed. As the concentration of the molecules increases, the amount of adsorption will rise, and the changes will be more significant. Since (especially in the visible range) the optical conductivity of the graphene layer is dependent on its density function and the amount of hopping energy of the graphene flakes, any change in these parameters will change the layer’s optical features. Additionally, as the dielectric constants of the WBC and RBC components are different, their role in these optical changes will be different and their detection via the suggested bio-chip would be possible.

It is also worth noting, as just a single monolayer of graphene has been considered on each wall, the large non-zero imaginary dielectric constant of graphene cannot cause a significant damping effect on the probe light intensity. Sreekanth et al. [14] have also proven that prior to that, the maximum sensitivity for a graphene-based biosensor was observed when just single a graphene layer was considered in 1D-PC. Additionally, it was recently shown that the graphene-based PC-biosensor performs more sensitively whenever it is driven by visible light sources (compared to the IR ones) [30]. Then, when we try to optimize the bio-chip response in this wavelength range, it is not only in line with that finding but also makes it possible to reduce the bio-chip size into nanometer levels. Another advantage of using graphene in the suggested sensor is related to the extra tunability in the sensor’s performance (that the graphene layer causes). Tuning the specific properties of graphene usually takes place using electrostatic or magneto-static gating or doping [19,20,21]. However, one more effective approach might be the mechanical tension method because, although graphene is a flexible material that can easily be rolled to fabricate zero or one-dimensional materials (similar to fullerene and nanotubes, respectively), it is one of the strongest two-dimensional materials that has been found yet. One more approach to tune its optical and electrical features is to adjust its band structure under controlled strain [32]. Strain can result in some significant quantum effects that might control the physical properties of graphene [33]. To the best of our knowledge, studying the effect of linear (uniaxial) strains on the sensitivity of a graphene-based bio-sensor has not been reported before, and this research is the first study in this regard.

### 2.3. Theoretical Approach

Second order partial differential equations, named as wave equations, are the fundamental formulas that are utilized to analyze the propagation of an electromagnetic wave, theoretically in both uniform and layered structures [34,35]. The homogeneous form of these equations is considered as:

(1) $$\left(v_{ph}^{2}\nabla^{2}-\frac{\partial^{2}}{\partial t^{2}}\right)E(or\;B)=0$$

where $\nu_{ph}=1/\sqrt{\mu \varepsilon }$ is speed of light in a medium with permittivity and permeability values of *ε* and *µ*, respectively. Spatiotemporal relations for electric and magnetic components of an electromagnetic wave (EMW) impinging at an arbitrary angle to a periodic structure might be extracted using Equation (1). Additionally, taking all of the boundary conditions into consideration, as well as the necessary continuity for tangential components of the fields across the consecutive adjacent layers, it is possible to calculate the coefficients of the related phasors in the extracted formulas [36,37]. Two components of the EMW, at the input and output interfaces of each layer, could be connected by a 2 × 2 transfer matrix for isotropic layers, by taking the dynamical and propagation matrices for each layer into concentration [38,39,40]. Thus, the tangential components of the electric and magnetic fields at the incident and exit end of the total crystal could be related to the product of the transfer matrices of different layers, i.e., for an ideal crystal made of the N periods of a unit-cell we have:

(2) $$\left[\begin{array}{l} E_{0}\\ H_{0} \end{array}\right]={\left\{\prod_{l}\left(M_{Dl}M_{Pl}\right)\right\}}^{N}\left[\begin{array}{l} E_{N}\\ H_{N} \end{array}\right]={\left\{\prod_{l}\left(\begin{array}{l} \;\cos(k_{zl}d_{l})\;\;-\frac{j}{p_{l}}\sin(k_{zl}d_{l})\\ -jp_{l}\sin(k_{zl}d_{l})\;\;\cos(k_{zl}d_{l}) \end{array}\right)\right\}}^{N}\left[\begin{array}{l} E_{N}\\ H_{N} \end{array}\right]$$

In our structure, the index ‘*l*’ denotes the layers of ZnS, Ge, graphene sheet, bio-cell, or any additional involved layers. $p_{l}$ equals to $k_{zl}/\omega \mu_{0}$ for TE and $k_{zl}/\omega \varepsilon_{0}\varepsilon_{l}$ for TM polarizations. Please take note that for nonmagnetic and isotropic materials, ε is considered a constant and *µ* = 1. However, in the case of anisotropic uniaxial materials, such as graphene stripe, it is described with a tensor [19,41,42]. For a graphene sheet in the x-y-plane, the diagonal elements of the permittivity tensor are non-zero and given by $\varepsilon_{G}=\left(\varepsilon_{G,t},\varepsilon_{G,t},\varepsilon_{G,⊥}\right)$. Taking into consideration the fact that a normal electric field cannot excite any current in a two-dimensional material, the normal component of the permittivity could be considered as $\varepsilon_{G,⊥}=1$. However, the tangential component depends on many parameters, such as angular frequency (ω), thickness of the graphene layer ($d_{G}$), the permittivity of the vacuum ($\varepsilon_{0}$), temperature, and graphene chemical potential ($\mu_{c}$) in the form of [42,43]:

(3) $$\varepsilon_{G,t}=\varepsilon_{G,⊥}+j\frac{\sigma (\omega )}{\varepsilon_{0}\omega d_{G}}$$

The surface conductivity of graphene contains two terms, named intra-band ($\sigma_{\mathrm{int}ra}$) and inter-band ($\sigma_{\mathrm{int}er}$). Details of the equations that describe them are expressed in [17,31,32]. Furthermore, for TE polarization, the dispersion relation is considered as: $k_{zl}=\sqrt{k_{0}^{2}\varepsilon_{l}-k_{x}^{2}}$, in which $k_{xl}=k_{x}=k_{0}\sin\theta_{0}$. However, in the case of TM polarization, the relations of $k_{z\;\;ZnS}=\sqrt{k_{0}^{2}\varepsilon_{ZnS}-k_{x}^{2}}$, $k_{z\;Ge}=\sqrt{k_{0}^{2}\varepsilon_{Ge}-k_{x}^{2}}$, and $k_{z\;g}=\sqrt{k_{0}^{2}\varepsilon_{g,t}-k_{x}^{2}(\varepsilon_{g,t}/\varepsilon_{g,⊥})}$ are regarded for the ZnS, Ge, and graphene layers, respectively. It is clear that whenever the normal incidence is considered, we have $k_{x}=0$ and then $k_{g,z}=k_{0}\sqrt{\varepsilon_{g,t}}$ in both polarizations. For unstrained graphene, the energy dispersion of non-interacting quasiparticles around a Dirac point (*K*) is linear and its Hamiltonian is $H_{0}=\hbar \upsilon_{f}\sigma .q$ [41,44], where *q*, $\upsilon_{f}$, and σ are the wave-vector displacement from the Dirac point, the Fermi velocity, and the Pauli matric in the x and y directions, respectively. When low strain amounts are applied uniaxially to the honeycomb lattice, the Dirac point location shifts, without opening the gap. Thus, the effect of applying uniaxial low strain is the distortion of the Dirac cone into a cone with an elliptical section [41]. In this case, the perturbed Hamiltonian is *H* = *H*_0_ + *H_S_*, and the energy dispersion still remains linear. *H_S_* is defined as [44]:

(4) $$H_{S}=\varepsilon_{S}\left(\begin{array}{l} \;\cos^{2}\theta -\upsilon \sin^{2}\theta \;\;(1+\upsilon )\cos\theta \;\sin\theta \\ (1+\upsilon )\cos\theta \;\sin\theta \;\;\;\sin^{2}\theta -\upsilon \cos^{2}\theta \end{array}\right)$$

Here, θ, $\varepsilon_{S}$, and υ are the directions in which the strain is applied to the graphene sheet, with respect to the *x* axis, the strain modulus, and the Poisson’s ratio, respectively. The optical conductivity of the deformed graphene under linear regime is calculated by [41]:

(5) $$\sigma_{strain}(\omega )=\sigma_{g}(\omega )\left[1-2\tilde{\beta }(1+\upsilon )\varepsilon_{S}\cos(2\theta -2\varphi )\right]$$

where $\tilde{\beta }$ is considered as 1.1 and *φ* is the logarithmic derivative of the nearest-neighbor hopping parameter (at $\varepsilon_{S}=0$) and the angle, which quasiparticles the momentum that vector *q* makes with the x-axis. Transmission, the spectrum of the chip, could be calculated by elements of the transfer matrix via [38,39]:

(6) $$T=\left\{ \begin{array}{l} {\left|t\right|}^{2}\;\;\;(TE)\\ \frac{p_{0}}{p_{n+1}}{\left|t\right|}^{2}\;\;(TM) \end{array}\right.,t=\frac{2p_{0}}{(m_{11}+m_{12}p_{n+1})p_{0}+m_{21}+m_{22}p_{n+1}}$$

where $p_{0}$ is equal to $\left(\sqrt{\mu_{0}/\varepsilon_{0}})\cos(\theta_{0}\right)$ in the case of TE polarization and $\left(\sqrt{\mu_{0}/\varepsilon_{0}})/\cos(\theta_{0}\right)$ for TM. Additionally, $p_{n+1}$ is $\sqrt{\mu_{n+1}/\varepsilon_{n+1}}\cos(\theta_{n+1})$ and $\left(\sqrt{\mu_{n+1}/\varepsilon_{n+1}})/\cos(\theta_{n+1}\right)$ for the TE and TM polarizations, respectively. $\varepsilon_{0}$ and $\varepsilon_{N+1}$ are denoting the relative dielectric constants of the input and output planes.

## 3. Results and Discussions

### 3.1. One-Dimensional (ZnS/Ge)^N^ Multilayer Geometry

**Figure 2:**
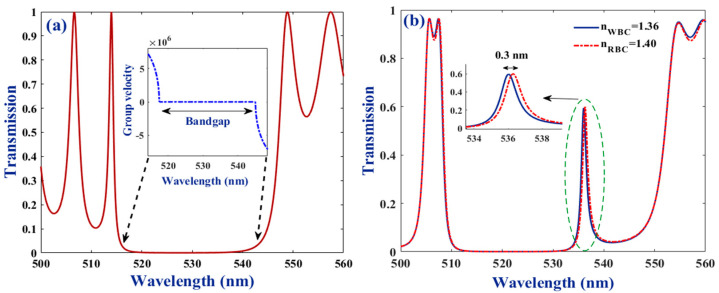
Transmission spectra for (**a**) an ideal perfect crystal made of 8 periods of (ZnS/Ge) unit-cells, (**b**) bio-chip, including 4 periods of unit-cells at each side of the single bio-cell. The inset in panel (**a**) indicates GV for the ideal crystal in the 518–545 nm wavelength range. The parameters of *θ* = 0°, *Ɛ_s_* = 0.2, *ψ* = 30°, *µ_c_* = 0.2 eV, d_Ge_ = 200 nm, d_ZnS_ = 330 nm, and d_bio_ = 90 nm have been considered in simulations.

The transmittance spectrum of a uniform multilayer structure made of alternating layers of ZnS and Ge slabs is illustrated in Figure 2a. It is clear that a wide band gap as large as ~27 nm is generated in the visible spectral domain when 8 periods of (ZnS/Ge) unit-cells are considered. The inset (in the same figure) represents data for the group velocity (GV) in the vicinity of the band gap spectral interval. Results show that GV possesses positive and negative values outside the band gap region and reaches zero in the gap range, in support of the behavior observed in transmittance spectrum. Panel (b) in Figure 2 illustrates the transmittance spectra for the structure with a geometry of (ZnS/Ge)^4^ bio-cell (ZnS/Ge)^4^ when it is filled with white and red blood components. Comparing the results in panels (a) and (b), it is clear that the resonant modes generated inside the stop band are due to the disruption of the structure symmetry and presence of the filled bio-cell. The resonant modes have almost the same intensity and are separated by just a 0.3 nm wavelength. Almost a total overlap was observed at other parts of these spectra. Thus, the structure can identify the WBC and RBC samples but its detectivity might not be that useful in practice. 

### 3.2. Methodology to Increase the Bio-Chip Performance

**Figure 3:**
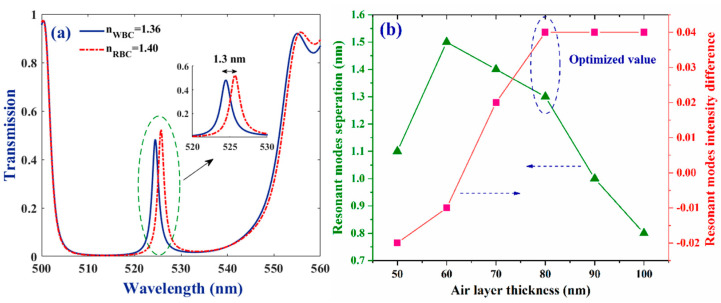
(**a**) Transmittance spectra of the bio-chip when bio-cell is capsulated in air slabs of d_air_ = 80 nm. (**b**) Dependence of the separation of the resonant modes’ central wavelength and their intensity difference (I_RBC_–I_WBC_) on the thickness of the air gaps. Number of periods of the unit-cells at the sides of the bio-cell is 3 and other parameters are considered the same, as presented in caption of Figure 2.

In order to improve the chip performance, the bio-cell was capsulated in-between two low refractive index layers. Air was selected as the material enclosing the bio-cell and plays the role of an intermediate layer for the light coming to (and exiting from) the cell. Figure 3 shows transmittance spectra for the novel configuration of (ZnS/Ge)^3^ air/bio-cell/air (ZnS/Ge)^3^. Note that in this geometry (compared to the structure discussed in the previous section), one period of ZnS/Ge unit-cells, at stacks located on both sides of the cell, was replaced by air. Then, the N value was considered as three periods. It is clear that, although the band gap experienced a blue shift, it still remained wide enough, and the sharp resonant modes were generated similarly inside the stop bands. Additionally, the separation amount of the resonant modes, related to the WBC and RBC samples, was much more remarkable, compared to what was observed in Figure 2b (~4.5 times). The size of the stop band was not different for the samples and an overlap was observed at other wavelengths of the related spectra. Taking these results into consideration, the optimal value for the size of the air layer was extracted by studying how the resonant modes are separated and their intensity amount differs as the size of the layer was changed. Panel (b) in Figure 3 shows these changes, in the range of 50–100 nm thicknesses. Increasing the air layer thickness from 50 to 60 nm, the resonant modes separated more. However, when increasing the size of the layer further, the resonant modes got closer, as at d_air_ = 90 and 100 nm their separation was even lower than what is for 50 nm thickness. On the other hand, when increasing the thickness of the air layer from 50 to 80 nm, the difference in the mode’s intensity amount increased continuously. However, for d_air_ ≥ 80 nm, the difference remains constant. It is also worth noting that the presented difference amounts were calculated for RBC intensity, minus the one for the WBC sample. Thus, as was observed, for thickness amounts of 50 and 60 nm, the intensity of the resonant mode for the WBC sample was more than the amount for RBC one. However, the situation changed at d_air_ ≥ 70 nm. According to the presented data, it seems that d_air_ = 80 nm is the optimum thickness, in which the resonant modes are well separated and their intensity difference is as remarkable as possible.

### 3.3. Effect of Period Number, Bio-Cell Thickness, and Light Polarization 

**Figure 4:**
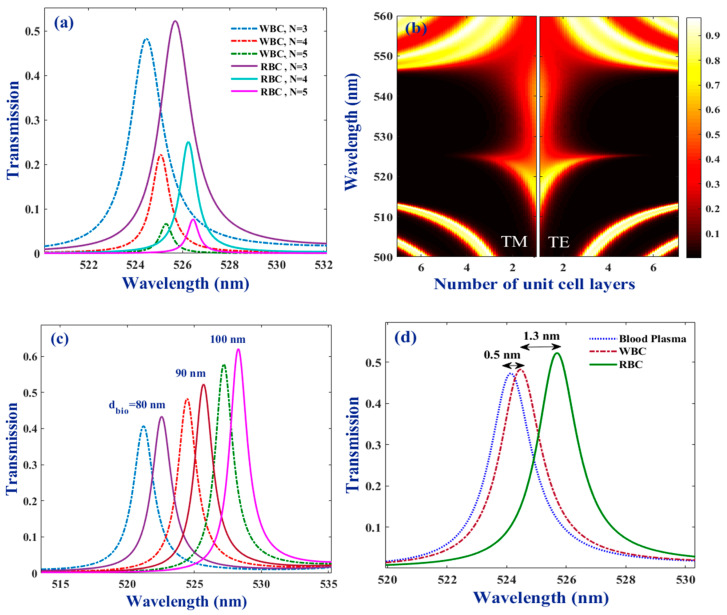
(**a**) Transmittance spectra for RBC and WBC samples when number of periods at each side of the bio-cell are N = 3, 4, and 5; (**b**) colormap of bio-chip transmittance versus the wavelength for the number of periods of 1 to 7 at each side of the bio-cell in the case of TE and TM polarizations; (**c**) transmittance spectra of the bio-chip for three different sizes of the bio layer—dashed lines belong to WBC sample and solid ones are for RBC; (**d**) resonant modes of WBC, RBC, and blood plasma samples—thickness of the air layer is 80 nm and other parameters are considered as presented in caption of Figure 2.

In the following, the dependence of the bio-chip performance on the structural and external parameters are explored systematically. Panels (a and b) in Figure 4 are indicating the role of the number of periods of unit-cells on the transmittance spectrum of the bio-chip with air layers of d_air_ = 80 nm. As is shown in panel (a), the resonant mode for the WBC sample was generated at lower wavelengths, compared to the mode for RBC sample when 3, 4, and 5 period numbers (at each side of the bio-cell) were considered. Additionally, for both samples, the intensity of the resonant modes reduced with increases in the period number. So, although the intensity of the resonant mode for RBC was ~7% more than the WBC sample at N = 3, at a period number of 5, they both had almost the same amount of intensity. Furthermore, a redshift took place for both resonant modes, by raising the value of the period numbers. Nevertheless, as the shift amount was almost equal for both samples, the separation of the resonant modes was nearly independent of the N value. Increasing the number of the periods of the unit-cells can clearly increase the probe light absorption amount, and thus, a reduction of the resonant mode intensity was as shown in Figure 4a. Additionally, as the number of the periods was increased, the effective optical path for the probe light increased. Furthermore, due to the novel extra layers, the number of side walls inside the crystal that light could be reflected from was also increased. Then, the number of waves going backward and forward in the crystal increased, and thus, it also increased the effective optical path length. Then, in order for the phase matching to take place, and thus, the resonant mode be generated, a redshift in the wavelength must be realized, according to the φ = k_zl_d_l_ relation. Figure 4a clearly illustrates this shift for the resonant modes of both the RBC and WBC samples by increasing the number of the periods.

Panel (b) in Figure 4 presents a colormap of the transmittance versus wavelength at the period numbers of 1 to 7 for two light polarization states of TE and TM. These data belong to when the bio-cell is filled by the WBC sample. Similar behavior was observed the for RBC sample and in order to avoid similar discussions it has not been reported in manuscript. It is clear that the size of the band gap was independent of the light polarization for all considered period numbers. Additionally, the central wavelength of the resonant mode was the same at two cases, i.e., independent of the probe light polarization state. The resonant mode was generated inside the stop band at the same wavelength. For both polarizations, at least three periods are necessary to reach a reasonable sharpness for the resonant mode. To further increase the period number, the mode becomes narrower until it disappears. However, it is also clear that at each period number, the width of the mode was, to some extent, more for the TM polarization and in this case the mode was suppressed to a large extent at N ≥ ~4. In the case of the TE polarization state, the resonant mode had a remarkable intensity, even at N = 5. Then, it was discovered that the suggested bio-chip had a proper response at both the TE and TM polarizations but using the TE state was better, in comparison. Thus, the TE polarization state is considered in the following, as the generation of the sharper resonant modes with higher intensities that do not suppress even up to N = 5 periods was possible in this case.

The results, illustrated in panel (c) of Figure 4, reflect that when increasing the thickness of the bio-cell (increasing the bio-fluid volume in the chip), a remarkable raise in their intensity (as was expected) was observed, besides the redshift for both resonant modes. Furthermore, the volume of the bio-fluid did not affect the mode’s generation sequence, and for all sizes of the bio-cell, the resonant peak for the RBC sample was generated at higher wavelengths, compared to the ones for WBC sample. The observed redshift in the transmittance spectra of the WBC and RBC samples might also be related to the increase in the effective optical path of the probe light, by increasing the size of the layer. Then, as was discussed above, for the effect of the period number, according to the phase relation of φ = k_zl_d_l_, when the size of the dl was increased, it was necessary for the resonant wavelength to be shifted to meet the phase-matching condition. Panel (d), in the same figure, presents resonant modes not only for the desired bio-samples (WBC and RBC) but also for blood plasma. It clearly shows that the suggested bio-chip can successfully separate the factors of WBC and RBC from their surrounding medium, so that the central wavelength of the considered biomaterials is different reasonably from blood plasma that can make it possible to easily identify the considered components of the medium with which they are immersed. In order to better evaluate the sensor performance, the quantitative values of the characteristics of the resonant modes for the selected bio-agents are tabulated in Table 1. According to the discussions presented above, the optimum values for dimensional characteristics of the bio-cell are considered as: 1 μm × 1 μm × 90 nm, and the volume of the sensing medium is held to be constant.

### 3.4. Incident Angle Dependency

**Figure 5:**
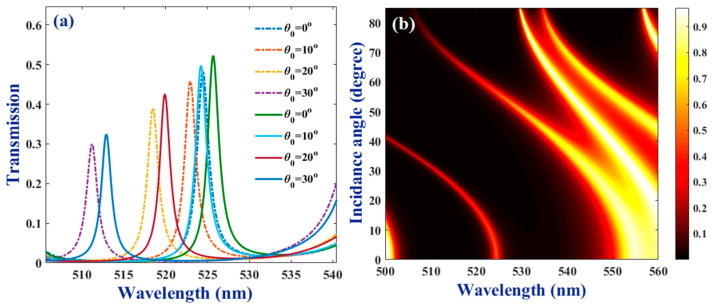
(**a**) Resonant modes of WBC (dashed lines) and RBC (solid lines) samples at different light incident angles of *θ* = 0, 10, 20, and 30°; (**b**) colormap of transmittance versus wavelength for incident angles of 0–85°. The thickness of the air layer is 80 nm and other parameters are considered, as presented in the caption of Figure 2.

The light incident angle was selected as another parameter for the simple tuning of the response of the suggested bio-chip. As a result, in the following, the dependence of the resonant mode characteristics on this angle are explored in detail. It was observed (Figure 5) that by increasing the incident angle from 0° to ~42°, peaks related to both the resonant modes of WBC and RBC, the samples experienced remarkable blue shifts. The amount of shift at low angles was much less, compared to higher ones. Besides, the deviation of the light incident angle from the normal direction results in the reduction of the resonant peak’s intensity amount. However, the reductions take place almost equally for both modes, which leads to the preservation of their intensity difference at all angles. However, their separation increases to some extent at higher degrees. Panel (b) of Figure 5 illustrates transmittance of the bio-chip filled by WBC sample versus wavelength for incident angle range of 0–85° in a colormap format. It is clear that changing the incident angle, not only the resonant mode inside the gap but also the limits of the stop band, as well as its size is affected. So that at normal incidence the resonant mode is generated at nearly middle of the stop band. However, increasing the angle it blue shifts followed by the blue shift that takes place for right-hand side limit of the gap. Additionally, the wavelength interval of the gap is seriously dependent on the incident angle. Finally, at *θ* ~42° the resonant mode reaches the left-hand side limit of the selected spectral range. However, at higher incident angles, once again, a wide stop band is generated in which the resonant mode, related to presence of the bio-sample, is very wide at first and close to the right-hand side boundary. Increasing the incident angle, its width reduces remarkably and its blue shifts, significantly.

### 3.5. Effect of Strained Graphene Layer

**Figure 6:**
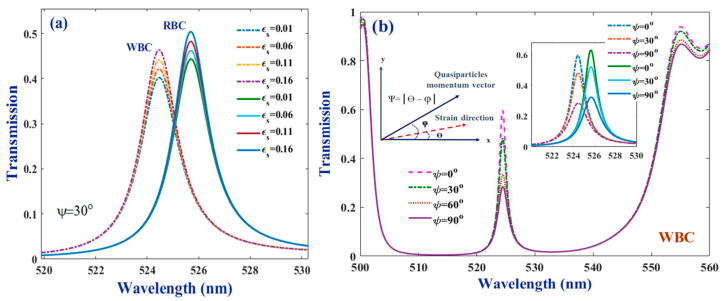
(**a**) Resonant modes of the WBC and RBC samples for different values of *Ɛ_s_* at *ψ* = 30^°^; (**b**) transmittance spectra of the WBC sample for different values of *ψ* degree, at a fixed value of *Ɛ_s_* = 0.2. Insets are presenting how *ψ* is defined and how resonant modes for the WBC and RBC samples depend on the *ψ* degree at *Ɛ_s_* = 0.2. Other parameters are considered, as presented in captions of Figure 2 and Figure 5.

**Figure 7:**
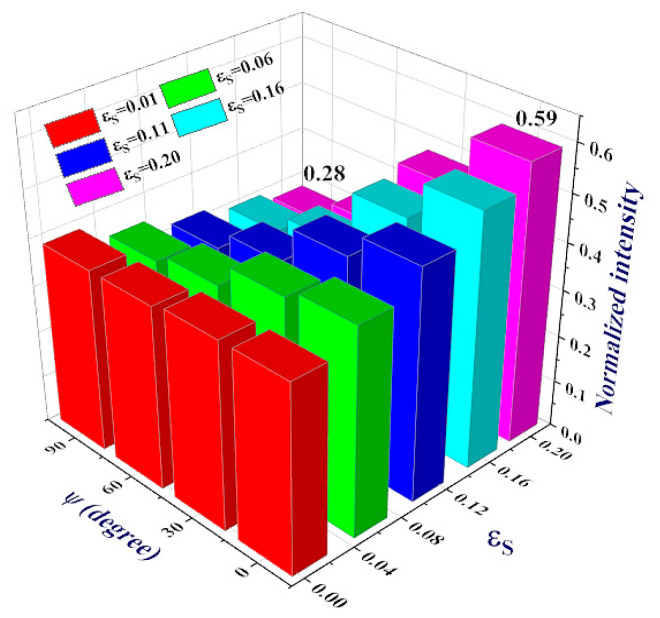
Values of the normalized intensities of the resonant modes for WBC sample, at different amounts of *Ɛ_s_* and *ψ* degrees. Other parameters are considered the same, as presented in caption of the Figure 2.

**Figure 8:**
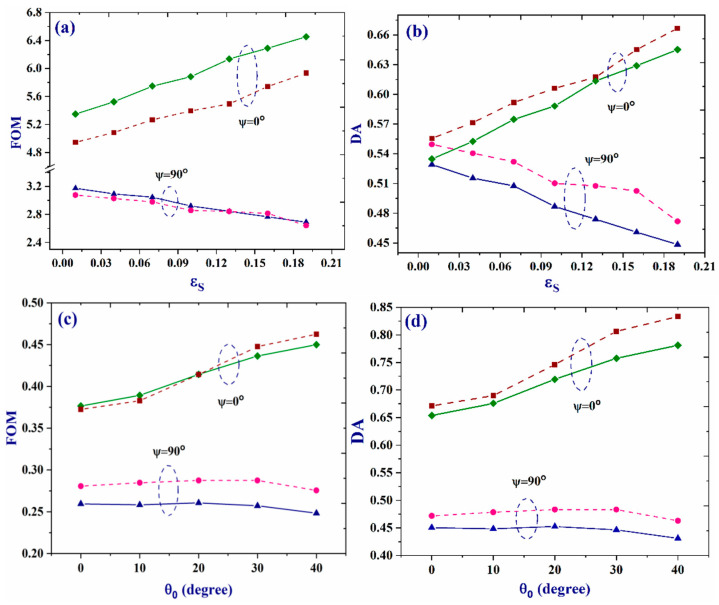
Values of figure of merit (FOM) (**a**,**c**) and detection accuracy (DA) (**b**,**d**) versus *Ɛ_s_* and light incident angle for two samples of WBC (solid lines) and RBC (dashed lines), at *ψ* = 0° and 90°. The values *θ*_0_ and *Ɛ_s_* are considered as 0° and 0.2 in panels (**a**,**b**) and (**c**,**d**), respectively.

Figure 6, Figure 7 and Figure 8 indicate how the resonant modes of the WBC and RBC samples change with the physical status of the coated graphene sheets at the side walls of the bio-cell. These results show that the optical response of the suggested bio-chip is dependent on not only the strain amount applied to the graphene sheets but also with the angle in which the stain is realized. According to panel (a) in Figure 6, when increasing the strain amount, the central frequency of both modes does not change. However, their intensity increases continually (almost equally for both samples) and their full width, at half maximum (FWHM), also reduces to some extent. Thus, the separation of the resonant modes and the difference in their intensity amounts remains constantly independent of the strain applied to the graphene sheets. However, they become more detectable as their intensity increases. Strain direction and how the *ψ* angle is defined, with respect to the direction of the quasiparticles momentum vector, are presented schematically in an inset in Figure 6b. The results illustrate that changing the angle of *ψ* (which, as described, contains information about the angle at which the strain is applied) the interval of the band gap region and its transmittance amount does not change (except for some possible small distortions at the upper corners of the left- and right-hand side limits of the band gap); the physical features of the band gap do not depend on the value of *ψ* parameter. However, increasing the *ψ* value the intensity of the resonant mode reduces remarkably, and its FWHM also increases. In other words, the sharpest resonant peak with the greatest amount of intensity belongs to the case of *ψ* = 0°. Similar behavior was observed in the case of the RBC sample (presented in inset at the same panel). Additionally, it is clear that by changing the *ψ* angle, resonant modes, related to the WBC and RBC samples, are changed in a way that their intensity difference, as well as their separation in wavelength, remains constant.

In order to increase our insight about the role of the *Ɛ_s_* and *ψ* parameters in the performance of the bio-chip, the intensity values of the resonant modes of WBC sample at different strain conditions are presented in Figure 7. It is revealed that both of these parameters might be utilized for tuning the bio-chip detectivity. However, a note should be taken that the dependencies are not uniform and linear. In other words, for *ψ* angles of 0 and 30°, by increasing the strain amount, the intensity of the resonant mode increases almost linearly. However, when the angle of *ψ* increases to the values of 60 or 90°, a descending behavior was observed in intensity versus the *Ɛ_s_* parameter. On the other hand, for low amounts of strain (*Ɛ_s_* = 0.01) the intensity of the resonant mode is nearly independent of the *ψ* angle (~39%). However, at further selected amounts of strain (*Ɛ_s_* = 0.06, 0.11, 0.16, and 0.20) the situation is different. At *Ɛ_s_* = 0.06, changing the *ψ* angle from 30 to 60°, a remarkable reduction jump in intensity was observed. However, when the angle increases from 0 to 30° or from 60 to 90°, the intensity reduces very smoothly. This is in support with the data presented in Figure 6b. However, applying more strain on the graphene sheets (at larger values of *Ɛ_s_*) mode intensity reduces more remarkably and continuously from *ψ* = 0° up to 90°.

The most remarkable descending jumps take place for the maximum selected amount of *Ɛ_s_* = 0.2. In summary, the maximum intensity of ~60% was observed at a maximum selected strain amount of *Ɛ_s_* = 0.2 and the minimum value of *ψ* = 0°. In reverse, the minimum intensity (28%) is realized at the same amount of strain but maximum angle of *ψ* = 90°, as has also been illustrated in Figure 7. Table 2 presents the quantitative values of the discussed parameters, to make it easy to evaluate the biosensor performance.

In order to even better evaluate the responsivity of the suggested bio-chip, the standard functional parameters are discussed at different circumstances in detail in the last step. For this purpose, the values of the figure of merit (FOM) and detection accuracy (DA = 1/FWHM) are extracted for two angles of *ψ* = 0 and 90°, various incident angles, and numerous strain parameters of *Ɛ_s_*. As changing the strain amount results in just the resonant mode intensity changings and not its central frequency, in order to extract detailed information, in the case of strain parameter, the FOM is defined as: (ΔI_mode_/Δ*Ɛ_s_*) × (1/FWHM). However, for the dependence on incident angle, we might consider FOM parameter as: angular sensitivity (Δλ_central_/Δ*θ*_0_), divided by resonant mode FWHM. The results are presented in Figure 8. Panel (a) illustrates variations of the FOM parameter versus strain for WBC and RBC samples. Two samples have similar behavior at two values of *ψ* angle. However, when *ψ* is considered as 90 degrees, a reduction in the FOM value, with increasing amounts of *Ɛ_s_*, was observed. Additionally, two samples have nearly equal values of FOM, at all *Ɛ_s_* values. However, when *ψ* is 0°, the samples are well separated by their FOM values at all amounts of strain and the chip gives higher FOM value for the WBC sample, compared to the RBC one. Furthermore, despite the case of *ψ* = 90°, the FOM value increases linearly for *ψ* = 0° by increasing the strain. However, it is also worth noting that the difference in FOM values for the two samples remained nearly constant with alterations of the *Ɛ_s_* amount. The dependence of the DA on the strain parameter of *Ɛ_s_* is also reversed at two degrees of *ψ* = 0 and 90°. It is worth noting that the DA values, presented in Figure 8, have been normalized in nanometer scale. In the case of DA and its dependency on the strain amount (similar to what was observed for FOM) a rising and descending behavior was observed for *ψ* = 0° and 90°, respectively. Values for RBC sample is more at all strain values and both *ψ* angles (except *Ɛ_s_* = 0.14 and *ψ* = 0), which means the FWHM values of the resonant modes for this sample are less at all cases. Additionally, for most values of *Ɛ_s_*, differences in the DA values for two samples are more detectable at *ψ* = 90°. Next, the dependencies of the FOM and DA parameters on the light incident angle are studied at two orientations of *ψ* = 0° and 90°. It is clear that values of both parameters are more in the case of *ψ* = 0°. Additionally, increasing the incident angle, the FOM and DA amounts increase for *ψ* = 0°, but their values are almost independent of the incident angle for the case of *ψ* = 90°. In more details, higher values of FOM and DA were observed for the RBC sample when *ψ* was 90° and, in both cases, the difference between values for this sample and WBC remained almost constant in the selected range of 0–40°. However, in the case of *ψ* = 0° (and for both samples), the values of the FOM and DA parameters increased with increases in the light incident angle. Besides, the values of the DA parameter were higher for RBC sample, compared to the amounts for WBC, at all incident angles. This means that, regardless of the angle in which the probe light is illuminated, sharper resonant modes are generated when the bio-chip is filled with the RBC sample. This behavior is more clear at *θ*_0_ = 30 and 40°. However, the FOM amounts of the RBC sample are only higher to some extent, at just *θ* = 30 and 40°. At lower angles, the FOM values do not differ that much for the two samples. 

In Table 3, the optimal data for 1/FWHM of the resonant modes (i.e., DA values, according to our definition) that have been reported by other researchers are compared with the values given by the suggested biochip at its optimum condition. Taking the detection accuracy (DA) as a criterion for this comparison into consideration, it is shown that the suggested sensor can perform well in bio-sensing applications. However, more experimental studies are necessary to further support the quality of the discussed biosensor.

## 4. Conclusions

A multilayer photonic bio-chip based on alternating slabs of ZnS and Ge semiconductors and a single bio-cell with graphene covered walls was suggested for the detection of blood components. The bio-chip is optimized to work with a probe light in the visible spectral domain. The results indicated that the isolation of the bio-cell with the air slabs has a significant effect on the performance of the chip. The detection capability of the chip was explored systematically by investigating how resonant modes of the WBC and RBC samples are separated and their intensity differs by changing the period number of the unit-cells, size of the bio-cell, width of the air slab, and the light incident angle, as well as its polarization state. It revealed that the suggested chip could perform well, even when the number of the periods of the unit-cells is as low as three at each side of the bio-cell and width of the sample cell is just 80 nm; in practice, it could be fabricated simply and can work well at very limited volumes of samplings. The optimum values for the air slab size and light incident angle were also detected as 80 nm and 40°, respectively. To increase our insight, concerning the tunability of the bio-chip response, special attention was also paid to the mechanical characteristics of the deposited graphene layers. The results show that the detectivity of the bio-chip is dependent on not only the amount of the strain applied on the graphene sheet but also the direction in which it is applied. We found that the best performance is realized when the *ψ* degree is zero and the *Ɛ_s_* parameter is 0.2. By following the issue quantitatively, it was (again) revealed that, independent of the light incident angle in the range of the 0–40°, the amounts of the FOM and DA parameters were higher for *ψ* = 0°, compared to *ψ* = 90°, for all strain amounts. Furthermore, it was found that resonant modes of the RBC samples were in (almost) all cases (different incident angles and strain amounts) sharper than the values measured for WBC ones at the same conditions. Thus, it seems that the suggested bio-chip, due to ease of its structural configuration and its highly convenient tunability in performance, might be considered a versatile, simple, and rapid tool for the label-free early detections of blood elements.

## Figures and Tables

**Table 1:** Difference between the intensity amount and FWHM values of the RBC and WBC samples at different values of the period number for L_bio_ = 90 nm and different values of L_bio_ at a fixed number of 3 periods.

L_bio_ = 90 nm	Intensity Difference (RBC − WBC)	FWHM Difference (RBC − WBC)	N = 3 (Periods)	Intensity Difference (RBC − WBC)	FWHM Difference (RBC − WBC)
N (Periods)			L_bio_ (nm)		
3	4%	1.2 nm	80	3%	1.4 nm
4	3%	1.1 nm	90	4%	1.3 nm
5	1%	1.1 nm	100	5%	1.1 nm

**Table 2:** Values of the intensity, central wavelength, and FWHM for WBC and RBC resonant modes at different amounts of the *ψ* and *Ɛ_s_* parameters.

White Blood Component (WBC)						
	*ψ* = 0°			*ψ* = 90°		
***Ɛ_s_***	**Intensity**	**Wavelength**	**FWHM**	**Intensity**	**Wavelength**	**FWHM**
0.01	40%	524.47	1.87 nm	39%	524.47	1.89 nm
0.07	45%	524.47	1.74 nm	35%	524.47	1.97 nm
0.13	51%	524.47	1.63 nm	31%	524.47	2.11 nm
0.19	58%	524.47	1.55 nm	28%	524.47	2.23 nm
**Red Blood Component (RBC)**						
***Ɛ_s_***	***ψ* = 0°**			***ψ* = 90°**		
0.01	0.44	525.69	1.80 nm	43%	525.69	1.82 nm
0.07	0.49	525.69	1.69 nm	39%	525.69	1.88 nm
0.13	0.55	525.70	1.62 nm	35%	525.70	1.97 nm
0.19	0.61	525.71	1.50 nm	32%	525.71	2.12 nm

**Table 3:** Comparison of the suggested sensor performance with the results of the previous sensors.

1/FWHM	Technique	Year	Ref.
0.62	SPR Based	2013	Sharma et al. [45]
0.057	SPR Based	2016	Quyang et al. [46]
0.098	SPR Based	2020	Keshavarz et al. [47]
0.5	PC Based	2021	Bijalwan et al. [3]
0.78 (WBC) 0.83 (RBC)	PC Based	Proposed here	Ghasemi et al.

## Data Availability

Not applicable.

## Sample Availability

Samples of the compounds are not available from the authors.
